# Rare earth-free magnetocaloric material Fe_82_Hf_6_Zr_7_B_4_Cu_1_ for high-temperature applications†

**DOI:** 10.1039/d5ra01759a

**Published:** 2025-05-09

**Authors:** Anjana Vinod, Arvindha Babu Diraviam, Manivel Raja Muthuvel, Madhuri Wuppulluri

**Affiliations:** a School of Advanced Sciences, Vellore Institute of Technology Vellore 632014 Tamil Nadu India; b Defence Metallurgical Research Laboratory Hyderabad 500058 Telangana India; c Ceramic Composites Laboratory, Centre for Functional Materials, Vellore Institute of Technology India madhuriw12@hotmai.com

## Abstract

The magnetocaloric effect (MCE) has garnered much attention in recent years, especially for rare earth (RE)-free magnetic materials. The increasing focus on these materials arises from their prospective uses in cryogenic magnetic cooling and elevated temperature environments. The study conducts a systematic experimental examination of a novel magnetocaloric material, Fe_82_Hf_6_Zr_7_B_4_Cu_1_ ribbons, primarily aimed at characterising their structural, magnetic, and magnetocaloric properties. X-ray diffraction (XRD) analysis confirmed the successful incorporation of hafnium in the Fe site of the Fe–Zr–B–Cu matrix. Furthermore, the magnetic properties of the ribbon were also investigated, yielding a Curie transition temperature of 678 K and a magnetic entropy change of 0.448 J kg^−1^ K^−1^ at 2.0 T. Notably, the relative cooling power and refrigeration capacity were determined to be 11.87 J kg^−1^ and 14.0 J kg^−1^, respectively, highlighting the potential of the material for high-temperature magnetocaloric applications. These findings collectively demonstrate that the novel Fe_82_Hf_6_Zr_7_B_4_Cu_1_ ribbon exhibits promising magnetocaloric properties, rendering it a suitable candidate for further investigation and potential applications in high-temperature magnetocaloric devices.

## Abstract 2

The magnetocaloric effect (MCE) has garnered much attention in recent years, especially for rare earth (RE)-free magnetic materials.

## Graphical abstract

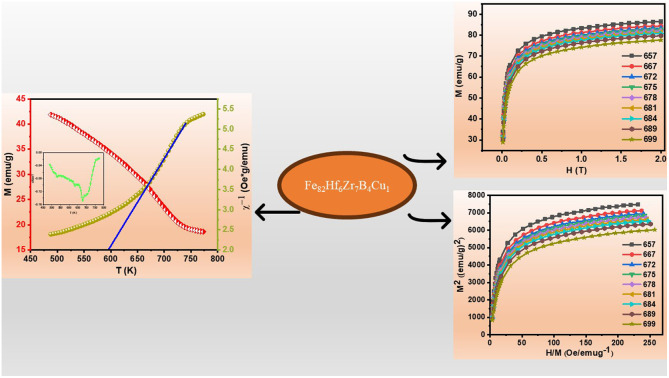

## 1. Introduction

In the realm of modern materials science, the magnetocaloric effect (MCE) is a phenomenon of utmost importance since it has the capacity to completely transform our approach to refrigeration and thermal control.^1^ The MCE, which is the reversible change in a material's temperature in response to an applied magnetic field, offers a viable substitute for traditional vapor-compression refrigeration.^2^ MCE's importance stems from its capacity to offer economical, eco-friendly, and effective cooling solutions for a variety of uses, such as air conditioning, refrigeration, and electronic cooling.^3,4^ Furthermore, innovative technologies like magnetic refrigeration systems, which have the potential to drastically lower energy use and greenhouse gas emissions, can be developed more quickly with the MCE.^5,6^ Moreover, because it offers a distinct viewpoint on the thermodynamic and magnetic characteristics of materials, the MCE is of great relevance for basic scientific research. In general, the field of MCEs is dynamic and rapidly changing, with the potential to revolutionise the way we approach refrigeration and thermal management.^7^

An intricate thermodynamic phenomenon, the MCE is the outcome of the interaction between the magnetic and lattice degrees of freedom within a material.^8^ The mechanism of the MCE is presented as follows: once a magnetic field is applied to a ferromagnetic material, the magnetic moments of the atoms align, resulting in a decrease in magnetic entropy.^9^ An increase in lattice entropy is associated with a decrease in magnetic entropy, as the vibrational motion of the lattice is promoted by the aligned magnetic moments.^10^ As a result, the material's overall entropy remains constant, despite the varying distribution of entropy between the magnetic and lattice degrees of freedom. When the magnetic field is removed, the magnetic moments become arbitrary, which leads to a decrease in lattice entropy and an increase in magnetic entropy. A reversible temperature shift is the defining characteristic of the MCE, which is the result of the entropy change being reversed.^6^ The MCE amplitude is contingent upon the substance's intrinsic magnetic and thermodynamic properties, the temperature of the substance, and the intensity of the magnetic field.^11^

Magnetocaloric materials are essential for numerous applications, and a thorough comprehension of the MCE process is essential for their design and development.^12^ Due to their exceedingly high Curie transition temperatures (*T*_C_), magnetocaloric materials have the potential to revolutionise a diverse array of industries and applications, surpassing the temperature of room air.^13^ Efficiency in the operation of these materials at elevated temperatures facilitates the development of innovative refrigeration systems, heat exchangers, and thermal management solutions for extreme environments.^14,15^ These materials are capable of operating reliably in demanding applications due to their high *T*_C_ values, which enhance their thermal stability.^16^ Magnetocaloric materials with *T*_C_ values exceeding 500 K (ref. 17) may be employed in industrial processes that entail high temperatures, such as the cooling of chemical reactors, heat exchangers, and furnaces.^16^ In addition, these materials can be employed in the cooling of high-temperature electronics, propulsion systems, and thermal protection systems, as well as in other advanced aerospace applications.^7^ In addition, magnetocaloric materials with exceptionally high *T*_C_ values can facilitate the development of innovative cryogenic refrigeration systems, superconducting applications, and quantum computing systems.^18^

The unique combination of high *T*_C_ values, robust thermal stability, and significant magnetocaloric effects of these materials renders them an appealing choice for a wide range of applications, including energy harvesting, thermal energy storage, and advanced thermal management systems.^19^ For the development of refrigeration systems that are compact, efficient, and environmentally friendly, magnetocaloric materials with high *T*_C_ are indispensable.^1^ A wide range of applications, including refrigeration, air conditioning, and electronic cooling, are suitable for these materials as a result of their high *T*_C_ values, which enable them to operate efficiently near to its *T*_C_.^20,21^ The high *T*_C_ values also result in a more efficient MCE of the magnetic moments, which are also more thermally stable.^22,23^ In addition, magnetocaloric materials with high *T*_C_ values can be implemented in applications that require a high thermal gradient.^24^ Among the applications of magnetocaloric materials with high *T*_C_ values are superconducting applications, electronic cooling systems, and aerospace and outer space applications.^25^ Generally, the development of magnetocaloric materials with high *T*_C_ values is essential for the advancement of environmentally favourable, compact, and efficient refrigeration systems. This has the potential to transform a varied array of industries and applications.^26^

In order to advance sustainable, cost-effective, and environmentally friendly technologies, it is imperative to develop rare earth-free magnetocaloric materials.^16^ Traditional magnetocaloric materials are significantly dependent on rare earth elements,^27–31^ which are not only expensive and subject to price fluctuations, but also pose significant environmental and social concerns as a result of their extraction and processing.^32,33^ Alternatively, magnetocaloric materials that are devoid of rare earths provide a more environmentally favourable and sustainable alternative, which facilitates the creation of innovative applications in the fields of energy harvesting, refrigeration, and advanced magnetic devices.^16^ Additionally, the diminished reliance on rare earth elements facilitates the development of more efficient and compact devices, mitigates the risks associated with price volatility, and improves supply chain security.^34^ In the analysis, the significance of rare earth-free magnetocaloric materials is their capacity to transform a variety of industries, such as aerospace, energy, and healthcare, by offering a more sustainable, cost-effective, and efficient solution for magnetocaloric applications.

FeZrBCu alloys have emerged as promising candidates for rare earth free high-temperature magnetocaloric applications due to their distinctive combination of properties.^35^ These alloys are capable of efficient operation at elevated temperatures due to their high *T*_C_, which is typically greater than 600 K.^36^ The thermal stability of the magnetic moments is improved by the addition of Zr and B to the Fe–Cu alloy system, which leads to a more pronounced MCE.^37,38^ The MCE in FeZrBCu alloys is distinguished by a high refrigerant capacity (RC) and a substantial magnetic entropy change (Δ*S*_M_), rendering them appropriate for high-temperature magnetocaloric refrigeration.^39^ Additionally, the FeZrBCu alloys demonstrate a relatively low magnetic hysteresis, which enhances the overall efficacy of the magnetocaloric cycle and reduces energy losses.^40^ The high-temperature magnetocaloric properties of FeZrBCu alloys render them appealing for applications such as thermal energy harvesting, heat transfer, and high-temperature refrigeration.

Further investigation of compositional modifications has the potential to improve the performance of FeZrBCu alloys, which exhibit promising properties for high-temperature magnetocaloric applications. The substitution of hafnium (Hf) in the FeZrBCu alloy system is one such approach. Upon conducting literature review, we discovered that there is very limited research on Hf substitution in FeZrBCu alloys. The objective of our research is to address this void in knowledge by investigating the impact of Hf substitution on the Zr site at a higher concentration. This could potentially result in enhanced magnetocaloric properties and enhanced application opportunities.

In this study, the magnetocaloric properties of a novel material system, Fe_82_Hf_6_Zr_7_B_4_Cu_1_, with a high *T*_C_ value are investigated. The material system, which is based on a rare-earth-free alloy, exhibits a high *T*_C_ value and a large magnetocaloric effect. A detailed investigation of the material's magnetocaloric properties, including its temperature dependence, field dependence, and refrigerant capacity is presented.

## 2. Experimental procedure

Vacuum arc melting was employed to synthesise the Fe_82_Hf_6_Zr_7_B_4_Cu_1_ ingots. The melting process was initiated by the generation of an electric discharge between a tungsten electrode and the metal constituents in a water-cooled, dismountable copper crucible plate with trough-shaped recesses. A sample of Fe_82_Hf_6_Zr_7_B_4_Cu_1_ ingots was subjected to a rigorous arc melting process that involved four successive melting cycles, with each side of the sample being melted twice. In order to guarantee sample homogeneity and mitigate potential contamination, this procedure was executed in a controlled argon atmosphere. Approximately 20 g of the arc-melted alloy were fragmented and placed in a transparent quartz crucible. The wheel speed was calibrated to 3000 rpm. A pneumatically controlled crucible receptacle facilitated the precise movement of the crucible up and down, thereby enabling the sample to melt uniformly within the induction coil. The alloy was meticulously positioned in close proximity to the rotating wheel once it had reached a molten state. The molten alloy was then ejected onto the rotating copper wheel at a regulated pressure of 2 psi, enabling the quick solidification of the alloy into thin ribbons.

## 3. Characterisation techniques

The Pan-analytical X-ray diffraction system was employed to conduct X-ray diffraction (XRD) analysis of the prepared Fe_82_Hf_6_Zr_7_B_4_Cu_1_ ribbons. The XRD measurements were conducted at a scanning rate of 4° min^−1^ using a Cu Kα source with a wavelength of 1.5406 Å. The structural properties of the samples were detailed by recording the diffraction patterns over a 2*θ* range of 35°–100°. The melt-spun ribbons were subjected to differential scanning calorimetry (DSC) using a TA Instruments Discovery SDT 650. A 90 μl alumina pan was used to meticulously place approximately 10–20 mg of each sample, which was subsequently heated from room temperature to 1523 K at a controlled rate of 20 °C min^−1^. KLA MicroSense EZ11 Vibrating sample magnetometers were employed to conduct temperature-dependent magnetisation measurements, during a temperature range of 657 K to 699 K. The measurements were performed with a constant magnetic field of 100 Oe. Isothermal magnetisation curves were measured at temperatures around the Curie transition temperature, with applied magnetic fields of up to 2.0 T.

## 4. Results and discussion

**Fig. 1:**
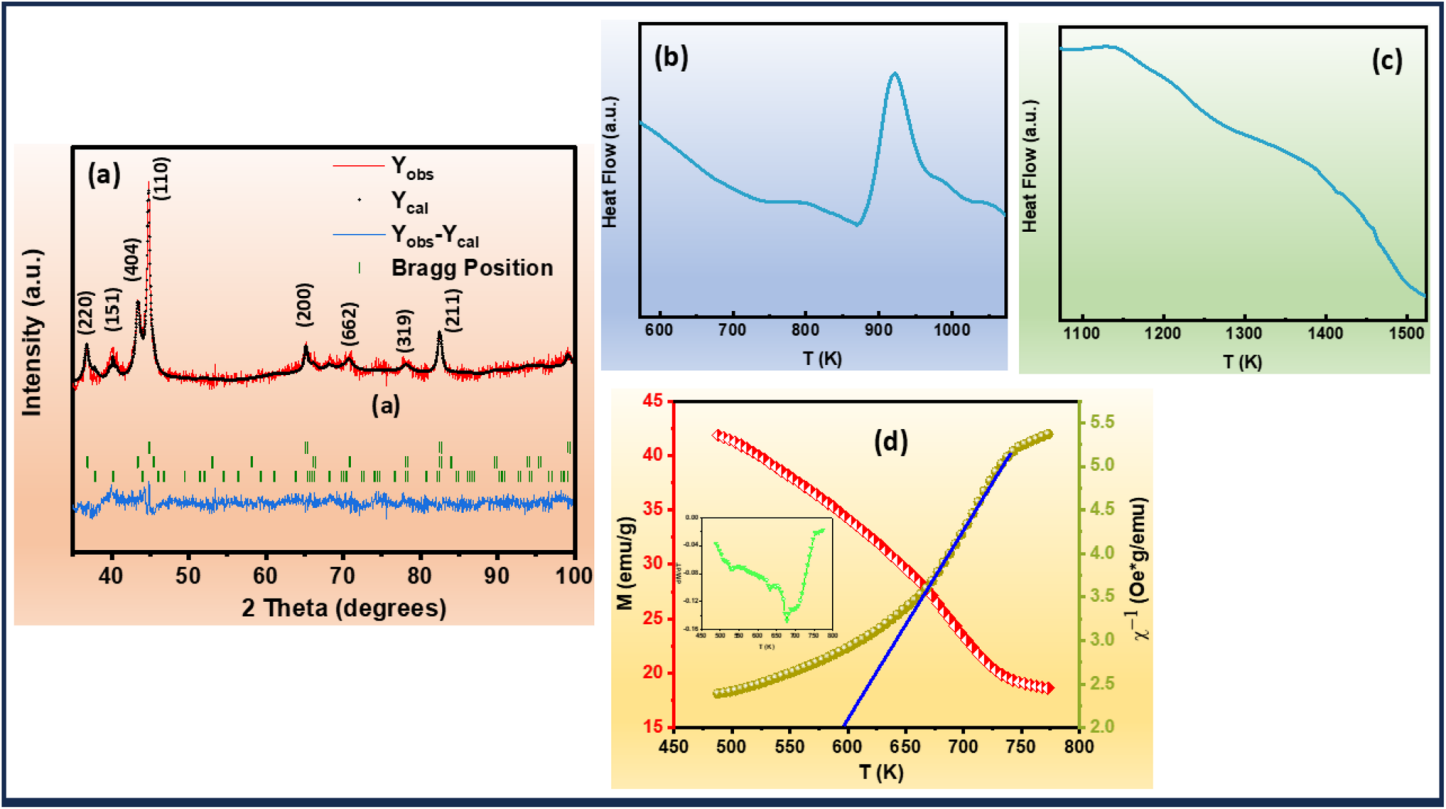
(a) The Rietveld refinement XRD analysis of Fe_82_Hf_6_Zr_7_B_4_Cu_1_ ribbons (b) and (c) DSC analysis of Fe_82_Hf_6_Zr_7_B_4_Cu_1_ ribbons (d) magnetisation *versus* temperature (left) temperature *versus* inverse susceptibility (right) and derivative of magnetisation *versus* temperature curve (inset) of Fe_82_Hf_6_Zr_7_B_4_Cu_1_ ribbons.

A comprehensive structural analysis of the alloys was conducted *via* X-ray diffraction (XRD) at room temperature, spanning the 2*θ* range of 35°–100°. To elucidate the phase composition and crystal structure of the ribbons, Rietveld refinement of the XRD patterns was performed, as illustrated in Fig. 1(a). The refinement revealed a complex multiphase composition, comprising three distinct phases: α-Fe, Fe_2_Zr, and Hf_2_Fe. Notably, the XRD patterns exhibited no detectable impurities, indicating high phase purity. The aforementioned observation depicted the successive hafnium (Hf) incorporation into the Fe-site of Fe–Zr–B–Cu matrix.

Differential scanning calorimetry (DSC) was employed to investigate the thermal characteristics of the Fe_82_Hf_6_Zr_7_B_4_Cu ribbons. The resultant DSC curves, presented in Fig. 1(b) and (c), provide valuable insights into the thermal properties of the alloys, including the liquidus temperature (*T*_l_) which is 1420.28 K and the initial crystallization temperature (*T*_x_) which is 881.24 K.^41^ Furthermore, the glass-forming ability (GFA) of the alloys was quantitatively assessed by calculating the GFA parameter, *α*, using eqn (1) and which is calculated to be 0.62.^42^1
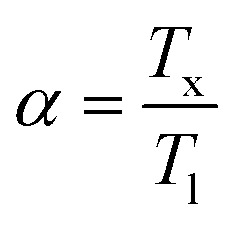


The composition of the alloy has a significant impact on the formation of a glassy phase in metallic systems. This can be qualitatively predicted by taking into account a number of critical factors. The enthalpy of mixing, which governs the thermodynamic stability of the alloy, the mismatch in atomic sizes among the constituent elements, which affects the topological and chemical short-range order, and the configurational entropy of the system, which influences the alloy's ability to form a disordered, glassy state, are among the factors. Researchers can acquire valuable insights into the alloying behaviour of metallic systems and identify promising compositions for glass formation by analysing these factors.

To elucidate the amorphous phase formation trend in the investigated multicomponent system, the glass-forming ability (GFA) was calculated using the *P*_HSS_ parameter, a predictive tool that effectively incorporates three crucial factors influencing glass formation. Traditionally, enthalpy effects in multi-component systems have been approximated by considering solely binary interactions between species, neglecting crucial elastic and structural contributions. To accurately determine the enthalpy of mixing (Δ*H*^C^) for liquid solutions, a comprehensive approach involves summing the individual binary interaction terms, as formulated in eqn (2).2
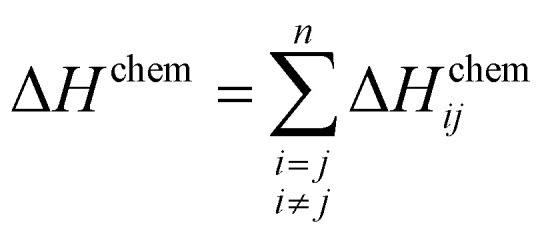
where the binary enthalpy of mixing Δ*H*^c^_AB_ is computed using the sub-regular solution model, disregarding short range order with *x*_A_ and *x*_B_ are atom fraction of elements A and B respectively and Δ*H*^interface^_A–B_ is the enthalpy of mixing for binary alloy at an infinite dilution of A in B, which is calculates according to Miedemma's semi-empirical model which is shown in eqn (3).^43^

3 Δ*H*^chem^_AB_ = *x*_A_*x*_B_(*x*_B_Δ*H*^interface^_A–B_ + *x*_A_Δ*H*^interface^_B–A_)

Mismatch entropy (Δ*S*_σ_) emerges as a critical factor, driven by atomic size disparities that inherently influence the alloy's thermodynamic stability and calculated by empirical relation given as in the eqn (4).^44^4
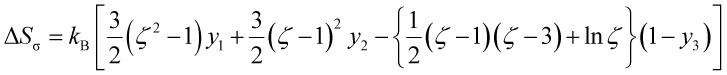
where 
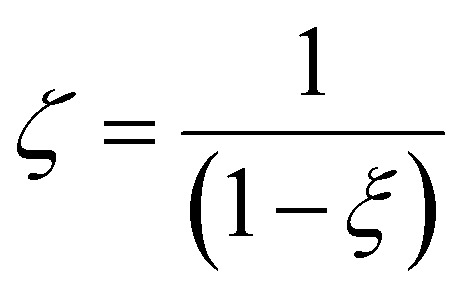
, *ξ* is the packing fraction, *k*_B_ is the Boltzmann's constant and *y*_1_, *y*_2_ and *y*_3_ are calculated by the eqn (5)–(8). 5
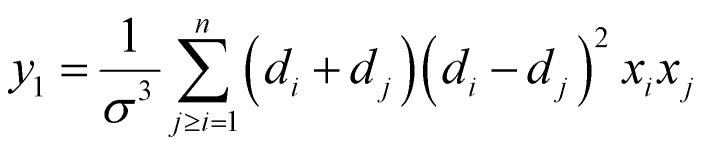
6
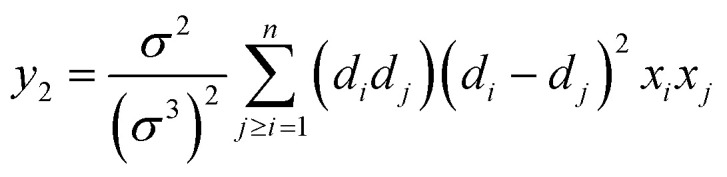
7
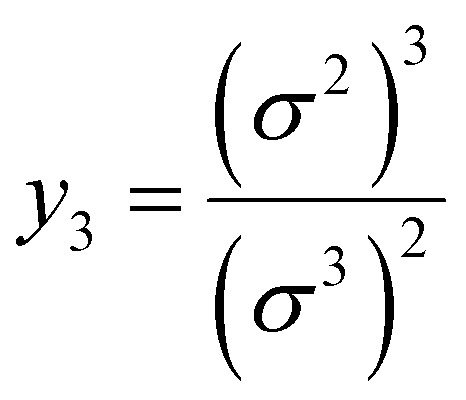
8
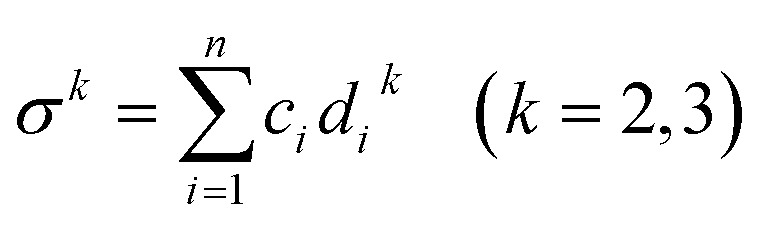
where *d*_*i*_ and *d*_*j*_ are the atomic diameter of the *i*^th^ and *j*^th^ elements. Configurational entropy (Δ*S*_C_) is exclusively determined by the atomic fractions of the constituent elements, independent of alloy-specific factors which is given by the eqn (9).9
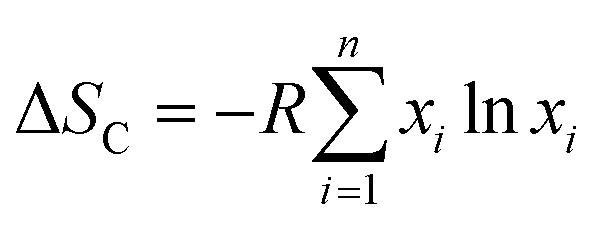
where *R* is the universal gas constant.

The glass forming ability of given samples are calculated using the eqn (10).10
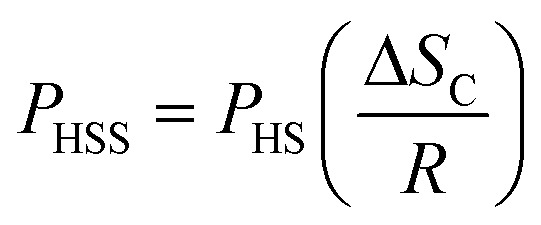
where *P*_HS_ represents the old GFA parameter, Δ*S*_C_ is the configurational entropy, and *R* is the gas constant. Furthermore, the *P*_HS_ parameter was determined using the eqn (11).11
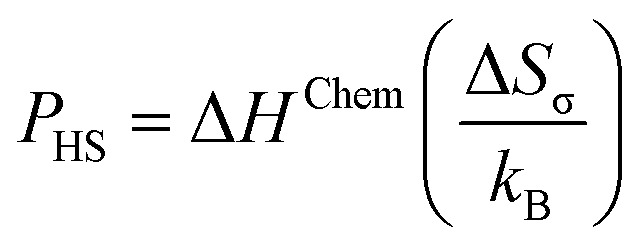
where Δ*H*^Chem^ denotes the enthalpy due to mixing, Δ*S*_σ_ represents the entropy due to atomic size mismatch, and *k*_B_ is the Boltzmann constant.^45^ By employing the comprehensive approach, the GFA of the given samples was systematically evaluated, providing valuable insights into the underlying mechanisms governing amorphous phase formation in these complex systems. The various physical parameters of Fe_82_Hf_6_Zr_7_B_4_Cu_1_ ribbons are presented in Table 1.

**Table 1:** Physical properties of Fe_82_Hf_6_Zr_7_B_4_Cu_1_ ribbons

Compositions	Δ*H*^chem^ (kJ mol^−1^)	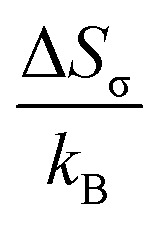	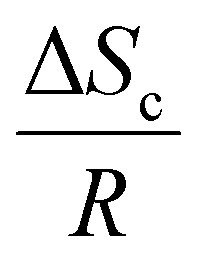	*P*_HSS_ (kJ mol^−1^)
Fe_86_Hf_2_Zr_7_B_4_Cu_1_	−14.99	0.09000	0.6900	−0.9980

The magnetization–temperature (*M versus T*) curve of the Fe_82_Hf_6_Zr_7_B_4_Cu_1_ ribbons were analysed under an applied magnetic field of 100 Oe to determine the Curie transition temperature. A ferrimagnetic–paramagnetic transition is indicated by the fact that the magnetisation decreases as the temperature increases, as illustrated in Fig. 1(d). The inflection-point method was implemented to precisely ascertain the magnetic transition temperature, which was determined by identifying the temperature that corresponds to the minimum derivative of the *M versus T* curve.^46^ This was accomplished by examining the 
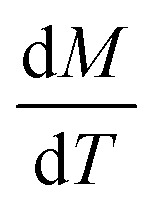
*versus T* plot, which is illustrated in the inset of Fig. 1(d). The Curie transition temperature value was precisely determined using the above-mentioned method, which is 678 K.

The Curie–Weiss law, which delineates the temperature dependence of magnetic susceptibility in paramagnetic materials, facilitates a thorough comprehension of the samples' magnetic behaviour. The relationship is mathematically represented as in the eqn (12).12
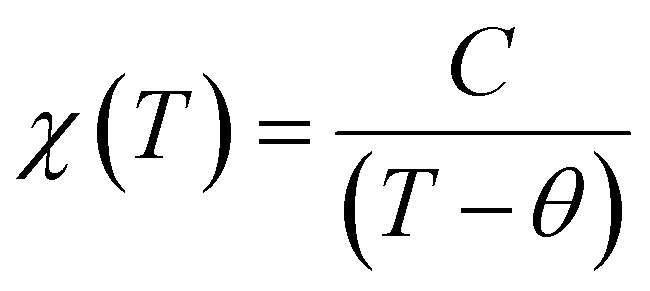
where *χ* denotes the magnetic susceptibility, *C* is the Curie constant, *T* is the temperature, and *θ* is the Curie–Weiss temperature.^47^ A fit to the experimental data (Fig. 1(d)) yields *C* = 1.13 emu K mol^−1^ and *θ* = 596 K. The experiment is carried out in a magnetic field of 0.05 T. A comprehensive analysis of the magnetic behaviour is provided by the temperature dependence of the inverse magnetic susceptibility, which is illustrated in right side of Fig. 1(d). The results demonstrate a high degree of agreement with the Curie–Weiss law.

**Fig. 2:**
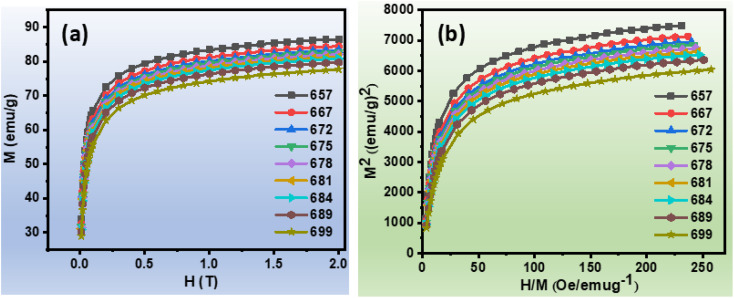
(a)Magnetisation *versus* magnetic field at constant temperature curve of Fe_82_Hf_6_Zr_7_B_4_Cu_1_ ribbons (b) Arrott curves of Fe_82_Hf_6_Zr_7_B_4_Cu_1_ ribbons.

The isothermal magnetisation curves of the ribbons Fe_82_Hf_6_Zr_7_B_4_Cu_1_ ribbons were measured over a wide temperature range to undertake a comprehensive investigation of their magnetic properties. The construction of comprehensive *M*–*H* curve was facilitated by the systematic increase in the magnetic field from 0 to 2.0 T. The first quadrants of the *M*–*H* curve (as shown in Fig. 2(a)) were employed to assess the variations in magnetic entropy as a function of temperature and magnetic field.^48^ Arrott plots were employed to conduct an extensive investigation of the alloy's magnetic transition, which serve as a valuable analytical tool for distinguishing between first-order and second-order transitions.^49^ The Arrott plot, depicted in Fig. 2(b), exhibits a positive slope at specific temperatures, suggesting a second-order magnetic transition. This discovery is especially noteworthy for magnetocaloric effect (MCE) applications, as second-order transitions are defined by minimal thermal and magnetic hysteresis, as per Banerjee's Criterion.^9^

13 *S* = *S*_electronic_ + *S*_lattice_ + *S*_magnetic_

According to eqn (13), a system's total entropy (*S*) is made up of three main parts: electronic entropy (*S*_electronic_), lattice entropy (*S*_lattice_), and magnetic entropy (*S*_magnetic_). However, because the contributions from electronic and lattice entropy are small, the isothermal entropy change (Δ*S*) can be used as a trustworthy approximation of the total entropy in some situations.^50^ The calculation of Δ*S* using the magnetisation isotherms *M*(*H*,*T*) shown in Fig. 2(a) is made possible by this approach.

Thermodynamic theory states that the eqn (14).14
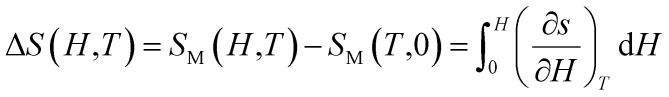
which is derived from the Maxwell relation depicted in the eqn (15).15
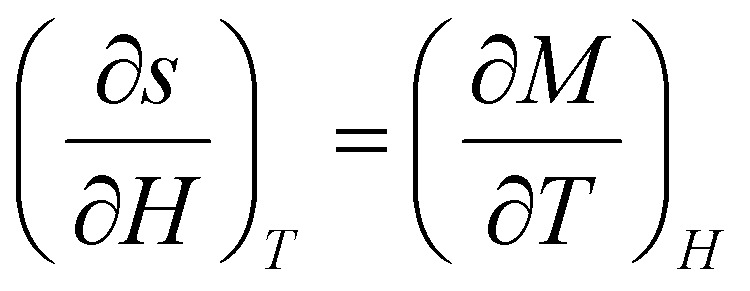
can be used to compute the isothermal field-induced magnetic entropy change (Δ*S*_M_) from 0 to *H*_max_. The isothermal magnetic entropy change can be accurately calculated by integrating the magnetisation isotherms, providing important information on the material's thermodynamic characteristics which is shown as in the eqn (16).^50^16
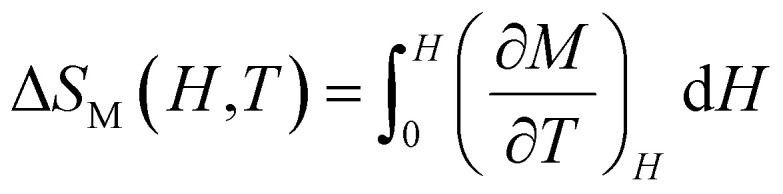


Moreover, it is possible to depict it as at discrete temperature intervals like in the eqn (17).^51^17
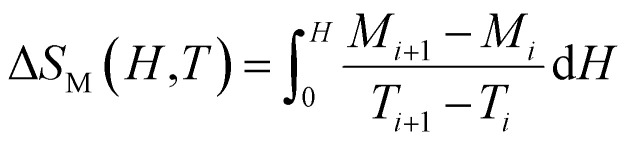


The magnetic entropy change (Δ*S*_M_) of the Fe_82_Hf_6_Zr_7_B_4_Cu_1_ ribbons as a function of temperature was examined under various magnetic fields from 0–2 T as given in Fig. S1.† This allowed for the analysis of the field dependence of Δ*S*_M_ by linearly fitting the ln(Δ*S*_M_) *versus* ln(*H*) plot and extracting the slope, denoted as the *n* exponent, at each temperature.^52^*n* exponent for the Fe_82_Hf_6_Zr_7_B_4_Cu_1_ ribbons is illustrated in Fig. S2† with a value of 1.02 observed near the Curie transition temperature (*T*_C_). The Δ*S*_M_*versus T* data at a magnetic field of 2.0 T is distinguished by the presence of a broad apex in the Δ*S*_M_(*T*) curve for Fe_82_Hf_6_Zr_7_B_4_Cu_1_ ribbons, which is centred near the Curie transition temperature (*T*_C_). This observation suggests a strong correlation between the temperature corresponding to the apex of the Δ*S*_M_(*T*) curve (*T*^peak^) and *T*_C_. Furthermore, the field dependence of Δ*S*^peak^_M_ was examined, which demonstrated a progressive increase as the magnetic field increased. The Δ*S*^peak^_M_ values for Fe_82_Hf_6_Zr_7_B_4_Cu_1_ ribbons were 0.448 J kg^−1^ K^−1^, under a field change of 2 T.

Fe_82_Hf_6_Zr_7_B_4_Cu_1_ stands out due to its exceptionally high Curie transition temperature of 678 K, surpassing most previously reported Fe-based alloys as shown in the Table S1.† This makes it an ideal candidate for high-temperature magnetic refrigeration applications, where materials with lower Curie transition temperatures often fail. Although its magnetic entropy change is moderate (0.46 J kg^−1^ K^−1^ at 2.0 T), this trade-off is acceptable for applications prioritizing durability and high operating temperatures, such as industrial waste heat management. The alloy's likely amorphous and nanocrystalline microstructure contributes to its thermal stability, soft magnetic properties, corrosion resistance, and scalability for fabrication. Overall, Fe_82_Hf_6_Zr_7_B_4_Cu_1_ unique combination of high Curie transition temperature, cost-effectiveness, and stability positions it as a superior material for high-temperature magnetocaloric applications, with potential uses in waste heat recovery and environmental cooling.

The refrigerant capacity (RC) of the Fe_82_Hf_6_Zr_7_B_4_Cu_1_ ribbons was assessed using the eqn (18).18
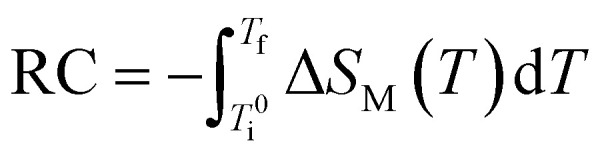


And relative cooling power (RCP) calculating by the eqn (19),^53^

19 RCP_FWHM_ = −Δ*S*^peak^_M_ × Δ*T*_FWHM_

where Δ*T*_FWHM_ denotes the temperature interval that corresponds to the full width at half maximum (FWHM) of the Δ*S*_M_(*T*)profile. The calculated RC values for Fe_82_Hf_6_Zr_7_B_4_Cu_1_ ribbons were 14.00 J kg^−1^ and RCP value were 11.87 J kg^−1^.

## 5. Conclusion

In conclusion, the Fe_82_Hf_6_Zr_7_B_4_Cu_1_ ribbons ribbon was successfully created using the melt spinning method, and its structural, thermal, magnetic, and magnetocaloric characteristics were thoroughly studied. The inclusion of hafnium (Hf) in the Fe site of the Fe–Zr–B–Cu matrix was validated by X-ray diffraction (XRD) research. The thermodynamic parameter *P*_HSS_, as determined by differential scanning calorimetry (DSC) study, was −0.998. It was discovered that the Curie transition temperature was 678 K and that the magnetic entropy change was 0.448 J kg^−1^ K^−1^ at 2.0 T. Relative cooling power and refrigeration capacity were determined to be 11.87 J kg^−1^ and 14.0 J kg^−1^, respectively. These findings imply that the novel Fe_82_Hf_6_Zr_7_B_4_Cu_1_ ribbons is a good candidate for high-temperature magnetocaloric applications due to its promising magnetocaloric applications.

## Conflicts of interest

There are no conflict to declare.

## Supplementary Material

RA-015-D5RA01759A-s001
