# Supplementary material for: Rare earth-free magnetocaloric material Fe82Hf6Zr7B4Cu1 for high-temperature applications
Source: RSC Adv. 2025 May 9;15(19):15310–7. doi: 10.1039/d5ra01759a (PMC12063070; doi:10.1039/d5ra01759a)
Supplement: RA-015-D5RA01759A-s001 [file RA-015-D5RA01759A-s001.pdf]

Supplementary Information (SI) for RSC Advances.
This journal is © The Royal Society of Chemistry 2025

Rare Earth-Free Magnetocaloric Material $Fe_{82}Hf_6Zr_7B_4Cu_1$ for High-Temperature Applications

Anjana Vinod[a], Arvindha Babu Diraviam[b], Manivel Raja Muthuvel[b] , Madhuri Wuppulluri[c*]

[a]*School of Advanced Sciences, Vellore Institute of Technology, Vellore 632014 Tamil Nadu, India*

[b]*Defence Metallurgical Research Laboratory, Hyderabad 500058 Telangana, India*

[c*]*Ceramic Composites Laboratory, Centre for Functional Materials, Vellore Institute of Technology*

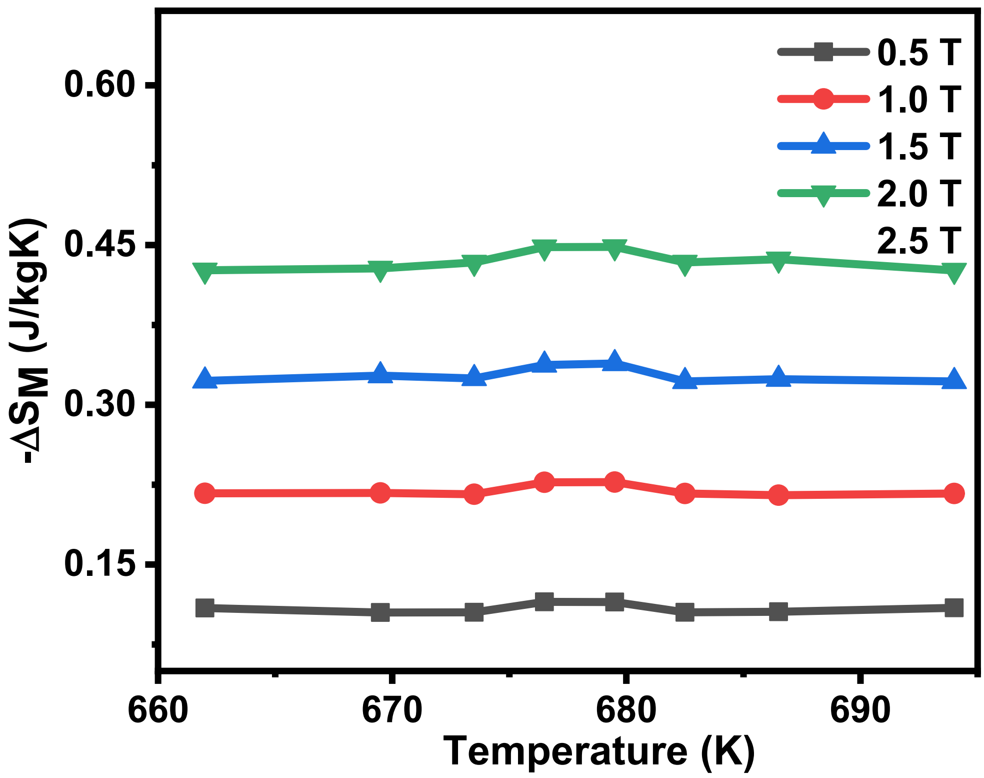

Fig.S.1. ΔS_Mversus T plot of $Fe_{82}Hf_6Zr_7B_4Cu_1$ ribbons.

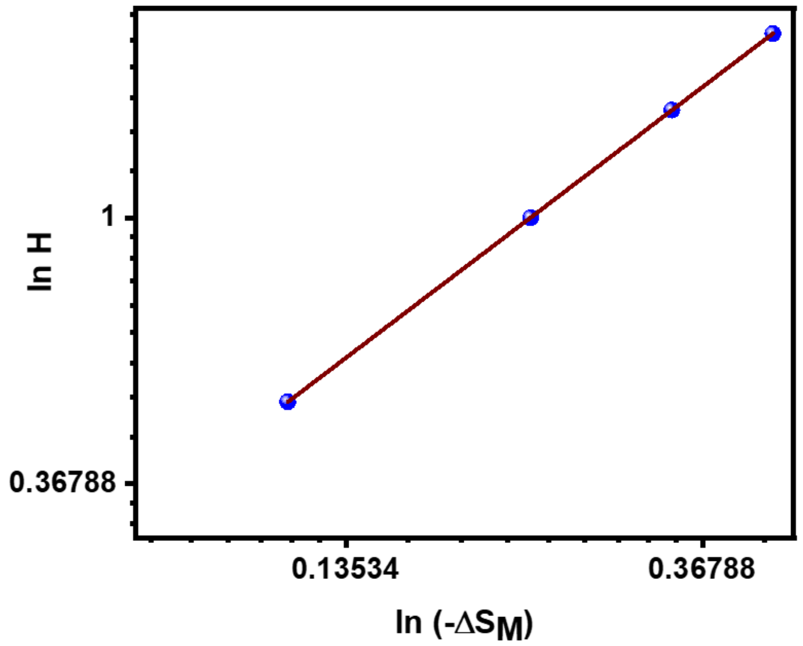

Fig.S.2. ln(ΔS_M) versus ln (H) plot of $Fe_{82}Hf_6Zr_7B_4Cu_1$ ribbons.

Table S1. Comparison of Magnetocaloric Properties of Fe-Based Alloys

**Alloy Composition**	T_C **(K)**	$-\Delta S_M$ **(J/KgK)**	**Application Temperature Range**	**Reference**
$Fe_{82}Hf_6Zr_7B_4Cu_1$	678	0.46 (2.0 T)	High-temperature	Present work
$Fe_{80}P_{13}C_7$	581	2.89 (2.0 T)	Medium-temperature	[1]
$Fe_{75}Co_5P_{13}C_7$	623	2.45 (2.0 T)	Medium-temperature	[1]
$Fe_{80}Cr_5B_2Zr_{10}Nd_3$	302	1.05 (2.0 T)	Room-temperature	[2]
$Fe_{86}Zr_8B_5Cu_1$	320	2.01 (2.5 T)	Medium-temperature	[3]
$Fe_{84}Zr_9B_6Cu_1$	328	1.81 (2.5 T)	Medium-temperature	[3]
$Fe_{82}Zr_{10}B_7Cu_1$	339	1.68 (2.5 T)	Medium-temperature	[3]
$Fe_{84}Zr_6B_{10}$	427	1.22 (1.4 T)	Medium-temperature	[3]
FeZrB(Cu)	~300-370	1.2-2.4 (0-8 T)	Room to Medium temperature	[4]
$Fe_{82}Ni_2Zr_6B_{10}$	465	1.38 (1.4 T)	Medium-temperature	[5]

References

1. Guo, J. *et al.* Effect of Co/Ni Substituting Fe on Magnetocaloric Properties of Fe-Based Bulk Metallic Glasses. *Metals (Basel).* **11**, 950 (2021).

2. Nguyen, H.-Y. *et al.* Structure, magnetic properties and magnetocaloric effect of

\(Fe_{81-x}Cr_{x+4}B_2Zr_{10}Nd_3\) rapidly quenched alloys. *HPU2 J. Sci. Nat. Sci. Technol.* **3**, 10–17 (2024).

3. Vinod, A., Diraviam, A. B., Muthuvel, M. R. & Wuppulluri, M. Tailoring the GFA, -ΔSM and RC of Fe86-2xZr8+xB5+xCu1 (x = 0,1& 2) metallic glass ribbons for magnetocaloric applications. *J. Non. Cryst. Solids* **650**, 123376 (2025).

4. Alvarez-Alonso, P. *et al.* High-magnetic field characterization of magnetocaloric effect in FeZrB(Cu) amorphous ribbons. *J. Appl. Phys.* **117**, 1–5 (2015).

5. Podmiljsak, B., Kim, J.-H., McGuiness, P. J. & Kobe, S. Influence of Ni on the magnetocaloric effect in Nanoperm-type soft-magnetic amorphous alloys. *J. Alloys Compd.* **591**, 29–33 (2014).
